# Leveraging Real-world Data to Increase Procedure Room Capacity: A Multidisciplinary Quality Improvement Project

**DOI:** 10.1097/pq9.0000000000000591

**Published:** 2022-09-23

**Authors:** Rachel Feldman, Daniel Low, Irina Gorbounova, Lusine Ambartsumyan, Lynn Martin

**Affiliations:** From the * Department of Anesthesiology and Pain Medicine, Seattle Children’s Hospital, Seattle, Wash.; † Department of Gastroenterology, Seattle Children’s Hospital, Seattle, Wash.; ‡ Department of Anesthesiology and Pain Medicine, Seattle Children’s Hospital, Seattle, Wash.

## Abstract

**Introduction:**

In the current healthcare climate, the financial strain created by COVID-19, limited resources, and case backlogs highlight the need to optimize operating and procedure room efficiency and maximize capacity. At Seattle Children’s, a clinical multidisciplinary team developed and implemented a data-driven protocol to improve efficiency in a high-volume gastrointestinal (GI) suite.

**Methods:**

Key process measures, including all case on-time starts and postanesthesia care unit length of stay, were extracted from the electronic medical record and presented as Statistical Process Control (SPC) charts. Clinicians’ performance was stratified by rational subgrouping to better understand variation in the system. We defined an expert clinician as one who performs beyond 3-sigma limits on funnel plot analyses. We developed clinical protocols based on expert clinician clinical practices. We gave clinicians dynamic, daily feedback on this family of measures through continuously updated SPC charts. This real-world data drove system and individual-level plan-do-check-act improvement cycles.

**Results:**

Despite significant external challenges over 2 years, procedure volume increased by approximately 25%, on-time starts improved by 36%, turnover time decreased by 34%, and postanesthesia care unit length of stay decreased by 15%. GI laboratory revenue increased by approximately 25% (independent of increased charges per procedure), representing the potential for a $2 million increase in annual revenue.

**Conclusions:**

A multidisciplinary clinical team improved efficiency metrics in a busy pediatric GI suite. Access to real-world data through continuously updated SPC charts enabled plan-do-check-act cycles that led to measurable improvement. This data access also served to sustain team motivation and engagement.

## INTRODUCTION

### Problem Description

Approximately 1,500 procedures are performed annually in our gastrointestinal (GI) suite, the vast majority being day-case upper or lower GI endoscopy cases. Statistical Process Control (SPC) charts, constructed using 22 months of historical electronic medical record (EMR) data from January 2018 to October 2019, revealed an average case delay of 23 minutes (with cumulative delays resulting in the room finishing late by an average of 32 minutes). In addition, there was large variability in provider workflow and anesthetic technique, which compounded the problems and resulted in an unpredictable in-room time and postanesthesia care unit length of stay (PACU LOS).

### Available Knowledge and Rationale

Increasing demand for OR and procedural facilities coupled with the need to contain costs has led hospitals to focus on efficiency. Endoscopy facilities, in particular, have unique process inefficiencies that lead to case delays despite relatively short procedure times.^1–4^ For example, a lack of flexibility to shift procedures to alternative locations and frequent proceduralist changes increase cumulative case delays.^2^ At our hospital, multidisciplinary team members, including GI nurses, anesthesiologists, and GI proceduralists, often complained about late and unpredictable work hours that did not seem to correlate with the number and complexity of cases scheduled. This concern led to a high staff turnover among nurses and physicians.

Meanwhile, over $40 billion of federal funds are invested in supporting EMR adoption in US hospital systems,^5^ generating increasingly large volumes of real-world data. Real-world data broadly refers to data collected in a nonrandomized controlled trial setting,^6^ including data generated as a product of routine care. The Institute for Health Improvement (IHI), one of the most influential forces in healthcare improvement in the United States, has been a proponent of SPC charts as the preferred method for making sense of these data to drive improvement in health systems.^7^ SPC charts allow for visualization of common cause variation (random noise inherent in the system) versus special cause variation (SCV) (significant signals not present in the system all the time). With the generation of large amounts of data from the advancement of EMRs, we have the opportunity to improve the efficiency and workflow of endoscopy suites and thereby increase procedure capacity and access.

### Specific Aims

Our goal was to evaluate and implement multidisciplinary workflow and anesthetic protocol changes to improve our endoscopy suite’s efficiency. We intended to have cases start and end on time reliably. Our primary aim was to:

1. improve all case on-time starts.

Secondary aims were to:

2. reduce PACU length of stay for all GI patients;
3. reduce room turnover time; and
4. improve the last case end time-delta (the difference between the actual end time of the last case and the scheduled end time of the last case).

## MATERIALS AND METHODS

### Context

Seattle Children’s (SC) is a quaternary referral academic medical center that serves pediatric patients (0–24 years of age) throughout Washington, Alaska, Montana, Idaho, and beyond. The GI endoscopy suite is physically embedded in the main operating room suite and staffed by a dedicated endoscopy nursing team. Most cases are day-cases that combine upper and lower endoscopies, therapeutic procedures, manometries, and liver biopsies. Independent anesthesiologists or anesthesiologists paired with a resident, fellow, or CRNA provide anesthesia. Patients and families are checked into a preoperative holding area and taken into the GI suite for their procedure under general anesthesia. Following their procedure, the patient will go to PACU phase 1 where they emerge from anesthesia. Once they have safely emerged, they are transferred to PACU phase 2, which is physically the same set of rooms as the preoperative holding area (dual function). From there, they are discharged home. Inpatients bypass phase 2 and go directly from phase 1 to the inpatient floor. SC has been capturing data electronically in their operating rooms and GI suite for over a decade, formerly on Cerner (Kansas City, Mo.) and more recently using Epic (Verona, Wis.).

### Intervention

Our approach began with a plan-do-check-act (PDCA) cycle, a tool described within the IHI’s improvement model. A PDCA cycle is used as a framework to guide improvement work.^8^

Baseline data over 22 months (January 1, 2018 to October 31, 2019), including 3,045 GI patients, were extracted and analyzed using AdaptX (Seattle, Wash.). We learned that the average case started 23 minutes later than its scheduled start time. The average PACU LOS was 79 minutes, with large variability, depending on which anesthesiologist cared for the patient. The average room turnover time was 35 minutes. The room finished on average 32 minutes later than the scheduled finish time.

**Fig. 1.**
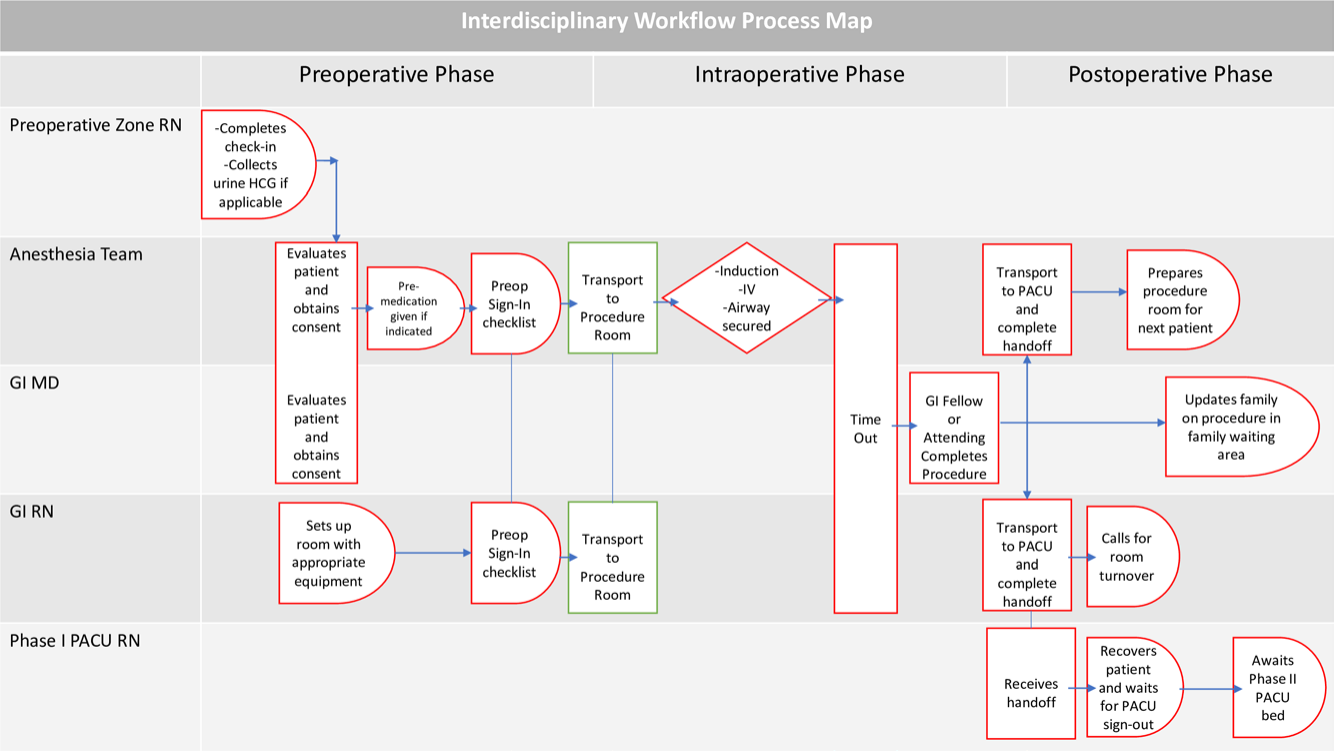
Process map illustrating presumptive causes of delay. The boxes highlighted in red show the targets of our interventions.

We engaged in process mapping around multidisciplinary team workflows to identify potential sources of delay (Fig. 1). For example, extended PACU LOS seemed related to (1) variable recovery times (related to variability in the combination and doses of medications used for anesthesia); (2) waiting for anesthesia sign outs; and (3) waiting for family arrival for discharge teaching. We worked to define “best practices” that would smooth areas of potential friction such as these.

#### Defining Best Practices for Anesthesiology

**Fig. 2.**
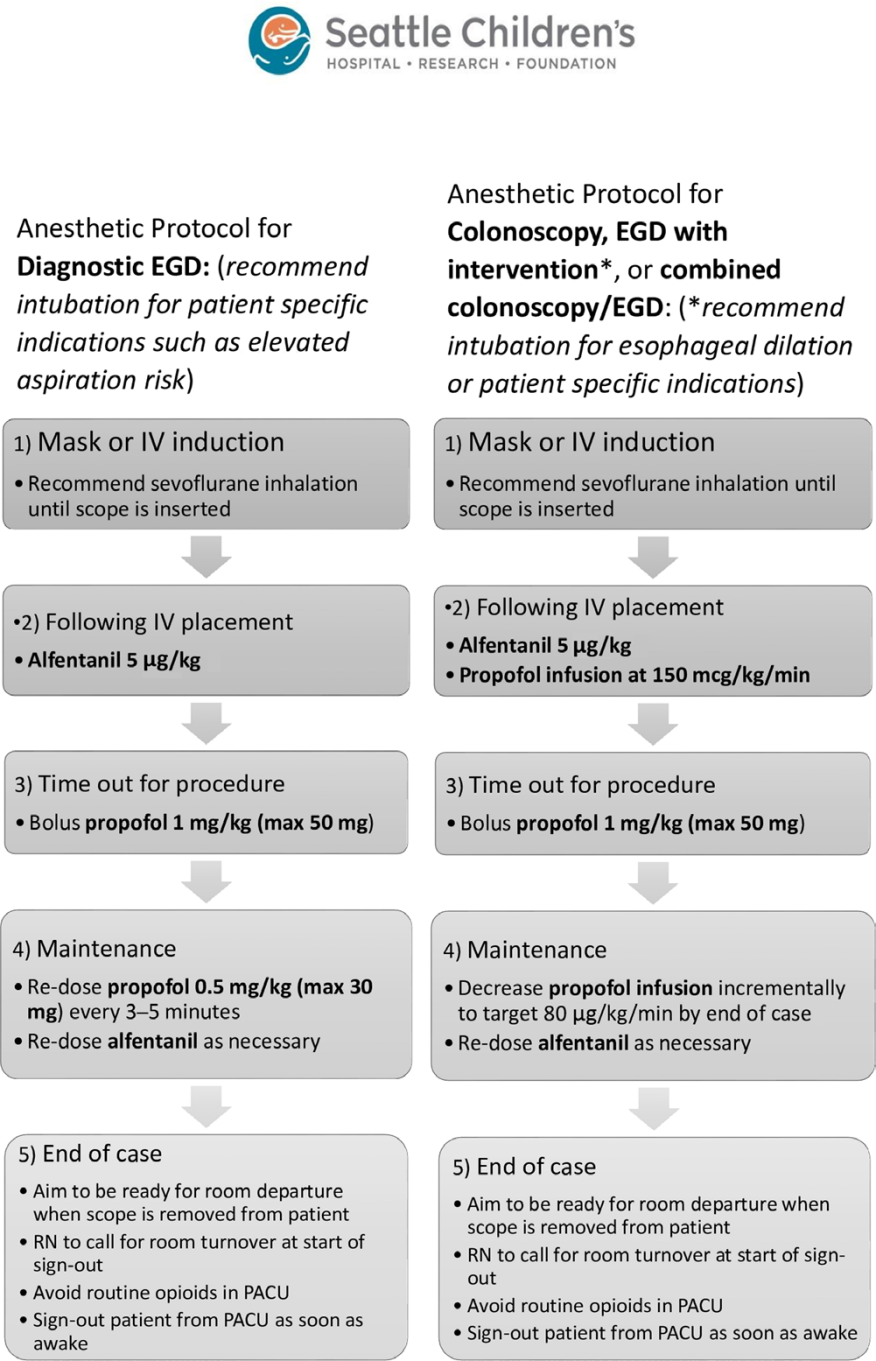
Suggested anesthetic protocol for upper endoscopy (esophagogastroduodenoscopy or EGD) and lower endoscopy (typically colonoscopy). Anesthesiologists were encouraged to adapt their practice as necessary based on clinical judgment.

Before November 1, 2019, there was no standard anesthetic protocol for GI procedures. Therefore, we used funnel plots of historical data from our institution that identified (1) anesthesiologists whose patients had the shortest PACU LOS following GI procedures and (2) anesthesiologists with the highest percentage of on-time starts. We interviewed and observed these individuals, then created a preliminary protocol based on their practices (Fig. 2). The protocol aimed to standardize clinical care, provide a safe and adequate depth of anesthesia, and allow for rapid and reliable emergence.

We selected a team of 8 anesthesiologists who helped refine the initial protocol over one month using real-time data reviews and consensus decision-making in weekly PDCA cycles.

#### Defining Best Practices for GI Proceduralists

We used funnel plots of historical data to identify proceduralists who had the shortest procedure times and whose cases had the smallest deviation from scheduled on-time starts. We interviewed these individuals regarding workflow practices and observed them clinically. The GI team committed to completing paperwork 10 minutes before scheduled procedure start times, communicating with the anesthesiologist when 5 minutes of procedure time were remaining, and visiting the next patient before updating the last patient’s family. Additionally, parents of patients undergoing only upper endoscopies waited in the preoperative room rather than returning to the surgery waiting area, so the proceduralist had less distance to cover between cases. Due to the longer duration of other procedures, keeping all families in the preprocedure rooms was not feasible as the rooms served the patients of multiple surgical services. At the start of each case, a timer was set by the circulator nurse for 5 minutes less than the anticipated procedure duration (15 minutes for upper endoscopy and 45 minutes for lower endoscopy) as a reminder to stay on track. In addition, the nursing team announced a 5-minute warning before the scheduled end time of the procedure to allow the attending to take over the procedure from the fellow when applicable.

#### Defining Best Practices for Nursing Teams

The nursing staff designed additional protocol components [see Figure 1, Supplemental Digital Content 1, http://links.lww.com/PQ9/A401, which displays the multidisciplinary job aid for gastroenterologists (“GI MD”), anesthesia providers, and periprocedural nursing teams (“GI RN, Zone RN”). The Yellow Zone is a multifunction patient care area for preoperative care and postoperative (phase 2) recovery, http://links.lww.com/PQ9/A401]. Furthermore, we instituted a daily morning huddle to promote a shared mental model for smooth workflow (see Figure 2, Supplemental Digital Content 1, http://links.lww.com/PQ9/A401, which displays the morning huddle checklist). This huddle included the anesthesiology team, the GI proceduralist, the GI nursing team, and the preoperative nursing team. The multidisciplinary team reviewed the schedule to identify challenging cases or potential causes for delays (such as the need for premedication or arranging for transport of inpatients to the preoperative area).

#### Initiating and Sustaining New Workflow

After the first 2 weeks, we presented the data to the anesthesiology and GI departments to acknowledge successes and challenges. The data were presented as SPC charts. Importantly, we instructed all physicians on how to continue accessing real-time metrics (updated daily) on the AdaptX platform. After the 1-month trial period was complete, other anesthesiology attendings were assigned to the GI suite. The protocol and workflow guidelines were emailed daily to this new staff and posted in the anesthesia work area. Each reminder email included a link to AdaptX so that providers could review current metrics. Following each anesthesiologist’s shift in the GI suite, we sent a separate email to elicit feedback on challenges encountered with the new protocol. In addition, we collected feedback from the GI physicians and nurses during discussions at monthly staff meetings. The primary author (R.F.) compiled all feedback and used it to inform future PDCA cycles.

After 6 months, we stopped distributing daily reminders and posted the protocol to a shared virtual folder for easy access. We emailed the anesthesiology and gastroenterology department updates on progress every 4–6 months. The update emails included recent SPC charts and a link to review real-time metrics. In addition, we posted guidelines in the preoperative zone for the nursing teams. Data were collected and reported for two years following protocol implementation.

**Fig. 3.**
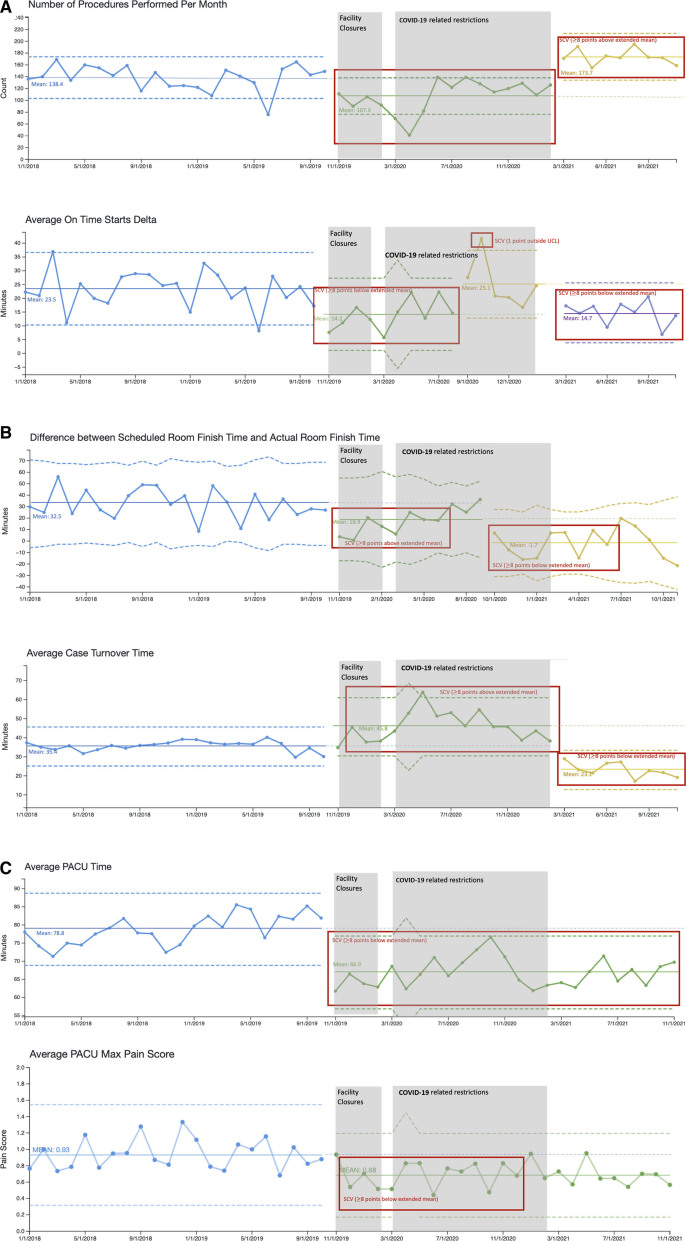
Baseline metrics for are displayed in blue for number of procedures performed per month (3A), average on time starts delta (3A), average difference between scheduled room finish time and actual room finish time (3B), average case turnover time (3B), average PACU time (3C), and average PACU maximum pain score (3C). Cohort 2 (green) started with initiating the multidisciplinary protocol (November 1, 2019). Subsequent cohorts and movement of the center line were based on patterns observed in the data. Red boxes highlight instances of SCV. Dashed lines represent upper and lower control limits (UCL and LCL), while dotted lines show extended means.

Our theory of improvement is summarized as a key driver diagram in Figure 3, Supplemental Digital Content 1, http://links.lww.com/PQ9/A401, which displays a key driver diagram summarizing our theory of improvement.

### Measures

(1) All case on-time starts (primary outcome) and (2) PACU LOS (secondary outcome) were initially chosen as outcome measures. We added (3) room turnover time and (4) the last case end time-delta during subsequent PDCA cycles as additional secondary outcome metrics to track improvement. We defined “All case on-time starts” as the average difference between scheduled and actual start times for all scheduled cases. We chose this instead of “percentage of cases starting on time” because it allowed us to quantify the impact of delays. Historically, first-case on-time starts (FCOTS) are chosen as a measure of perioperative efficiency^9^; however, there are several limitations associated with this measure, particularly the fact that it does not track inefficiencies that build up throughout the day.

We defined PACU LOS as the time of arrival to phase 1 to discharge from phase 2 (outpatients) or the time of discharge to the floor (inpatients).

We defined room turnover time as out-of-room time with the current patient (“wheels out”) to in-room time for the following patient (”wheels in”). “Last case end-time-delta” was defined as the difference between the scheduled finish time for the last case and the actual finish time for the last case. We found this to be more meaningful than the average daily delay from the scheduled start time because it reflected the ability of the team to get back on track if one unusual case caused all remaining cases of the day to get pushed back.

We used procedure time as a balancing measure. We chose this to ensure that the protocol did not lead to an inadequate depth of anesthesia, necessitating breaks to deepen the anesthetic. We defined procedure time as procedure start time to procedure finish time. Additional balancing measures included maximum pain score recorded and postoperative nausea and vomiting treatment rates in the PACU. Pain scores were documented by PACU nurses using one of three validated pain scales^10,11^ (faces, legs, activity, cry, consolability; faces pain scale-revised; or visual analog scale), depending on the age and developmental status of the patient. We converted the pain scores to an 11-point range (0–10) for analysis.

### Analysis

We plotted baseline and postimplementation data on SPC charts and used Shewhart’s theory of variation for data interpretation.^12^ Nonrandom variation due to special causes is called SCV. For example, improvement (or deterioration) from a protocol change would result in SCV. Standard SPC rules state that SCV can be identified by (1) a single point located outside the control limits, (2) a run of eight or more points in a row above or below the mean centerline, (3) two of three consecutive points located near the outer one-third of the control limit, or (4) six consecutive points increasing or decreasing. We set the upper and lower control limits at three sigmas.

### Ethical Considerations

The Institute of Medicine defines quality healthcare as safe, effective, patient-centered, timely, efficient, and equitable.^13^ This Quality Improvement work’s purpose was to continue providing safe and effective care while improving timeliness and efficiency, improving access to care for patients in need. As a Quality Improvement project that did not involve human subject research, IRB review and approval were not required.

## RESULTS

There was SCV for procedure volume (mean 138 to 174 patients per month, Fig. 3A); all case on-time starts (mean delay 23.5 to 14.7 minutes, Fig. 3A); case turnover time (mean 35.4 to 23.1 minutes, Fig. 3B); and PACU LOS (mean duration 78.8 to 66.9 minutes, Fig. 3C). Analysis of room finish times showed that postimplementation, scheduled cases finished an average of 1.7 minutes *before* the scheduled end of the day (typically 5 pm). In contrast, before protocol implementation, cases finished 32.5 minutes *after* the scheduled end of the day (Fig. 3B). Balancing measures, including procedure duration (mean 23.6 to 24.1 minutes, not pictured) and treatment of postoperative nausea and vomiting in PACU (stable at 3.9% of cases of cases, not pictured), did not show SCV. Maximum pain scores in PACU (another balancing measure) decreased (0.9 to 0.7, Fig. 3C). Figure 4, Supplemental Digital Content 1, which contains S charts for the corresponding Xbar charts seen in Figure 3, http://links.lww.com/PQ9/A401.

Facility-related closure of the GI laboratory occurred intermittently between November 13, 2019, to February 7, 2020. Procedures were performed in a remote hospital location, accounting for the initial decrease in procedure volume (Fig. 3A), decreased on-time starts (Fig. 3A), earlier room finish times (Fig. 3B, attributed to decreased volumes), and increased case turnover time (Fig. 3B). Starting March 2020, elective case cancelations due to the COVID-19 pandemic further reduced case volumes. On-time starts and room turnover time also worsened during this period due to (1) hospital policy that required all staff to wear a controlled or powered air-purifying respirator (CAPR or PAPR) for lower endoscopy cases and (2) expanded cleaning protocols. CAPR/PAPR requirements were dropped in mid-January 2021. Starting March 2021, rescheduling of previously delayed procedures increased. Process changes from PDCA cycles allowed on-time starts, on-time finishes, and case turnover times to improve despite increased case volumes that began March 2021. PACU LOS (Fig. 3C) improved with the initiation of PDCA cycles and stayed stable throughout the facility complications.

## DISCUSSION

### Summary

Real-time SPC charts allowed for meaningful improvement in efficiency metrics and increased case volumes without compromising patient safety. Real-time SPC charts facilitated direct feedback, encouraged adoption, sustained engagement, and enabled rapid adaptation. The results show that, on average, we performed 36 additional procedures per month without delaying room finish times, leading to increased hospital revenue. Financial data was obtained directly from the financial head of the GI department. The charge rate for GI procedures increased by approximately 10% between two 7-month periods pre- and postintervention (April 1, 2019–October 31, 2019, versus April 1, 2021–October 31, 2021), while total GI laboratory revenue increased by 35%. The projected increase in revenue related to improved efficiency would be approximately $2 million annually. In addition, an analysis of weekday overtime payments to GI nurses during the same periods showed a $5,000 decrease, representing potential cost savings of approximately $9,000 per year.

### Interpretation

Implementing workflow changes that impact multiple large groups of individuals requires a commitment to follow the protocols and participate in further PDCA cycles of improvement. By having multiple stakeholders involved, we provided consistent messaging to multiple teams. Some individuals had specific objections to the guidelines; for example, one anesthesiologist preferred to use dexmedetomidine as part of the anesthetic for a colonoscopy. We explained our technique for designing the protocol and acknowledged that it was not a requirement but a starting point to be adjusted based on judgment.

During the second week of our trial period, an unplanned facility-related closure led to the relocation of GI procedures to another hospital wing. This move required the GI team to work in an unfamiliar setting and required the anesthesia team to transport patients over a long distance for recovery. Our team adapted to this challenge by creating a transport checklist to promote a reliable and safe process and practice.

The COVID-19 pandemic significantly impacted caseload, workflow, and procedure times. New personal protective equipment guidelines required CAPR/PAPR use for all COVID-unknown patients and patients undergoing colonoscopy. Our team attempted to maintain other workflow components despite these significant new roadblocks to efficiency.

Due to the COVID-19 pandemic and the previously mentioned facility closures, case volumes were lower during early 2020 (particularly in March and April), which initially impacted the earlier room finish times. However, with increasing case volumes throughout the pandemic, we maintained the earlier room finish times. This result illustrates that interpretation of SPC charts must occur within the broader clinical context. Unlike data gathered from a clinical trial, these data reflect the “real-world” scenario with multiple internal and external variables at play daily. Without controlling each variable, quality improvement efforts can focus on consistently improving the system and evaluating its short-term and long-term impact.

Prior studies have used a similar methodology to define, interpret, and improve efficiency metrics in GI suites. For example, Palchaudhuri et al^4^ performed a thorough root cause analysis to identify causes of delay and design interventions that would decrease late inpatient endoscopic cases in an urban tertiary-care academic hospital. Tomer et al^14^ successfully improved on-time starts in a pediatric GI suite after identifying patient, equipment, and physician-related causes for case delays. Almeida et al engaged in direct observation and qualitative interviews to identify process inefficiencies that quality improvement initiatives could target.^1^ Although our methodology also included process mapping to identify causes of delay, we incorporated a unique approach to identify examples of positive deviance.^15^ Funnel plots allowed us to identify physicians with the best efficiency metrics. We observed their practices and incorporated their behaviors into our recommendations for best practices during PDCA cycles. Basing interventions on examples of positive deviance may have contributed to adoption and adherence.

### Limitations

We should tailor quality improvement work to improve efficiency for each unique setting and institution. Therefore, the individual protocol elements described above are not generalizable to other settings. Rather, the use of real-time data to drive PDCA cycles and engage multidisciplinary teams is a technique that can be adapted broadly for use by clinicians and administrators.

We focused primarily on four metrics, but these could not adequately capture the impact on our patients and teams. The limited number of metrics also did not allow for identifying specific elements responsible for room delays. Importantly, we did not capture our interventions’ impact on the experience of our team members as they adapted to a workflow with a new emphasis on efficiency. Finally, we did not thoroughly investigate the potential negative impact on fellow education. However, the fellows informally acknowledged that learning to balance education and production was a useful lesson for the future.

### Concluding Summary

“Efficiency” has different meanings to different stakeholders, and despite widespread acceptance of its importance, there is continued debate on the best ways to measure and improve it. Our project illustrates that (1) identification of positive deviance and (2) real-time data sharing are feasible tools to motivate multidisciplinary teams working toward improving efficiency. In addition, efficiency-focused quality improvement projects may benefit from prioritizing data literacy and democratization through data sharing.

### Disclosure:

Dr. Low is the Chief Medical Officer and founder of AdaptX, the software used to visualize and interpret data. The other authors have no financial interest to declare in relation to the content of this article.

## Supplementary Material


