# Supplementary material for: Leveraging Real-world Data to Increase Procedure Room Capacity: A Multidisciplinary Quality Improvement Project
Source: Pediatr Qual Saf. 2022 Sep 23;7(5):e591. doi: 10.1097/pq9.0000000000000591 (PMC10997288; doi:10.1097/pq9.0000000000000591)

Seattle Children's
HOSPITAL • RESEARCH • FOUNDATION

Anesthesia/GI/Nursing Job Aid

- **Daily Huddle** to occur 15 minutes prior to first case in the Yellow zone.
- Goal is to have the **same 2 rooms in the Yellow zone** (not guaranteed).
- **Families will not go out to waiting room** unless zone is busy and cannot accommodate them.
- **During time out**- GI RN will let attending know the start time for the case (for a reference if behind or on time).
- At start of case the GI RN will start **timer for the case time minus 5 minutes**.
- GI MD will **communicate to anesthesia provider when there are 5 minutes remaining** for the procedure.
- GI RN will **call for turn over before sign-out**.
- GI MD will speak to the following patient before updating the previous family. The following patient will be in the adjacent room and will be able to go into the GI suite while attending is talking with previous family.

Optimizing GI Room: Morning Huddle Checklist

When?	15 minutes prior to first case (typically 7:15a)
Where?	Yellow Zone Desk
Who?	GI attending (lead)<br>GI circulator nurse<br>Anesthesia attending<br>Pre-op zone nurse
1. Introductions<br><br>2. Review patient list<br><br>3. Any concerns?	Team introductions and exchange of contact info for the day<br><br>Identify single procedure EGD cases - discuss possibility of keeping family in the zone for these + others?<br><br>Open discussion

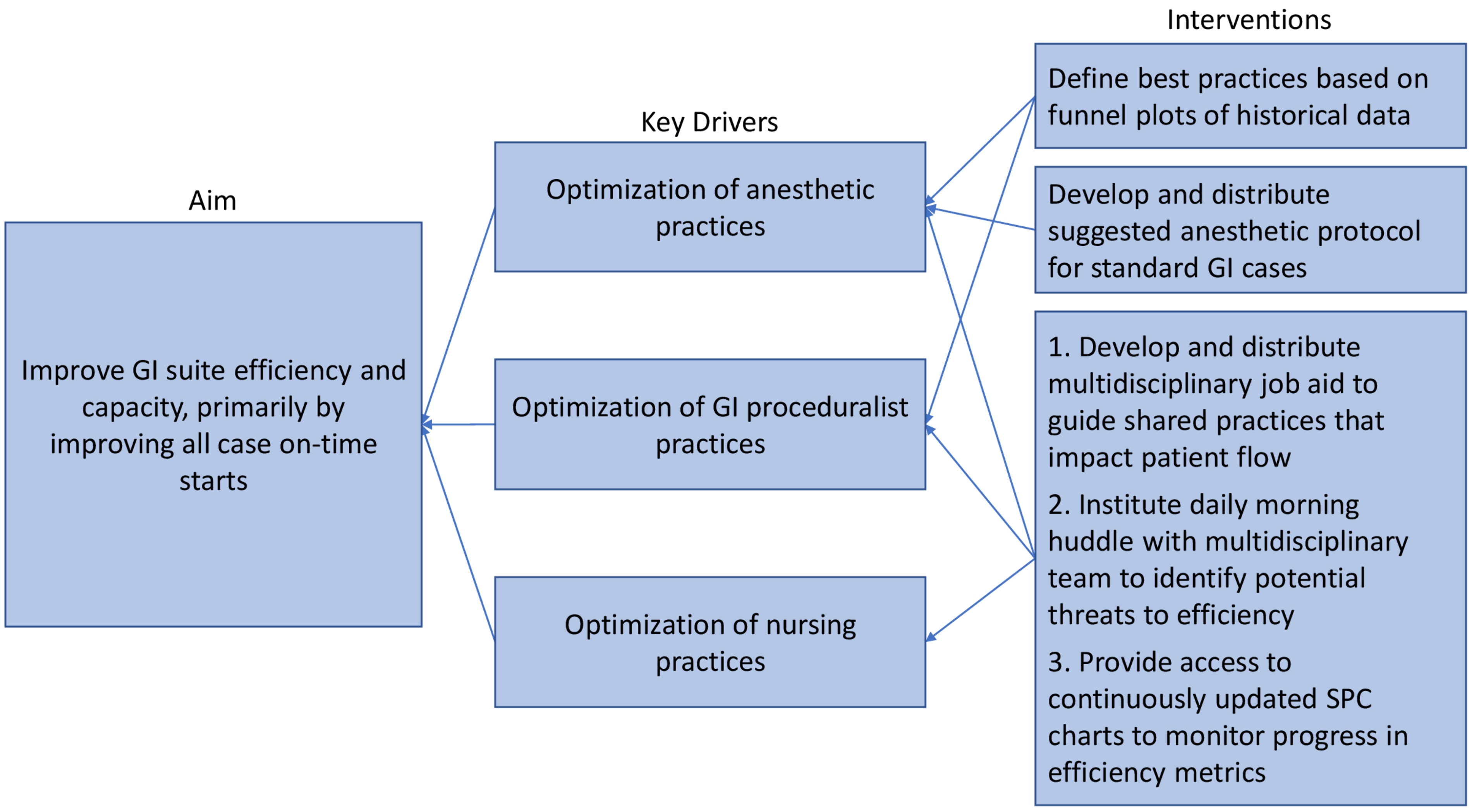
Aim
Improve GI suite efficiency and capacity, primarily by improving all case on-time starts
Key Drivers
Optimization of anesthetic practices
Optimization of GI proceduralist practices
Optimization of nursing practices
Interventions
Define best practices based on funnel plots of historical data
Develop and distribute suggested anesthetic protocol for standard GI cases
1. Develop and distribute multidisciplinary job aid to guide shared practices that impact patient flow
2. Institute daily morning huddle with multidisciplinary team to identify potential threats to efficiency
3. Provide access to continuously updated SPC charts to monitor progress in efficiency metrics

Standard Deviation of On Time Starts Delta *(See Figure 3A)*

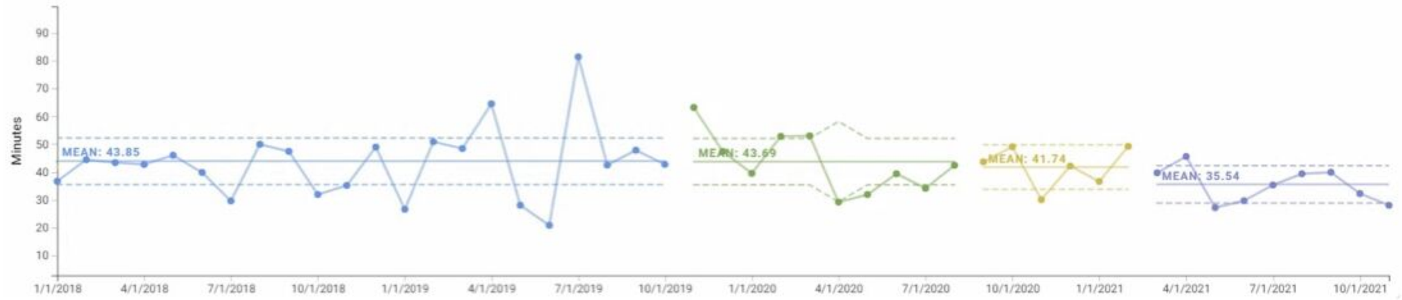

Standard Deviation of Last Case End Time Delta *(See Figure 3B)*

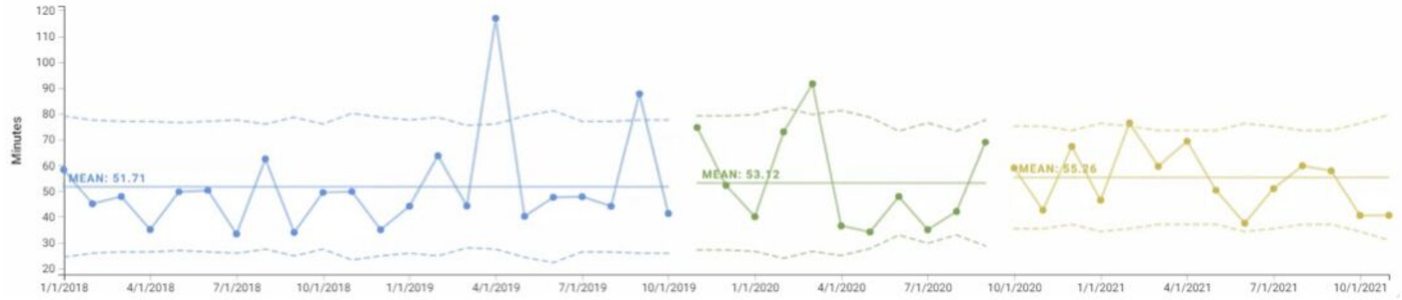

Standard Deviation of Case Turnover Time *(See Figure 3B)*

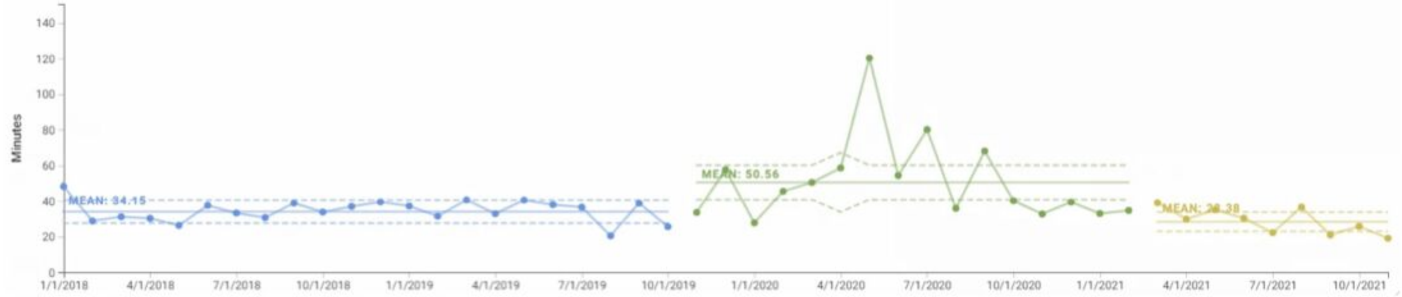

Standard Deviation of PACU Time *(See Figure 3C)*

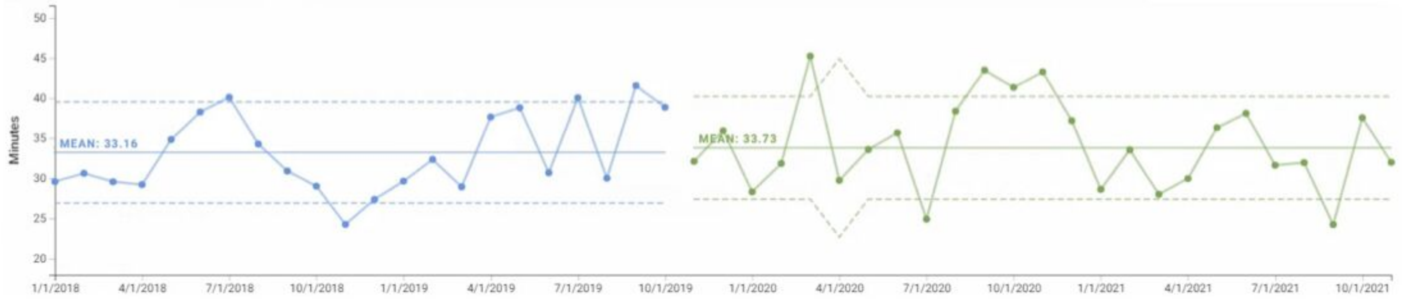

Standard Deviation of PACU Max Pain Score *(See Figure 3C)*

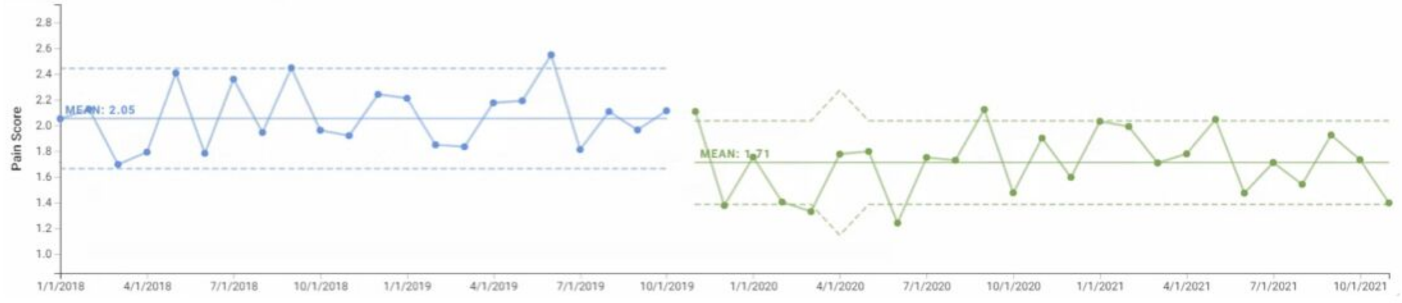

Supplement: Supplementary file 1 [file pqs-7-e591-s001.pdf]
